# Investigation into the Advantages of Pure Perovskite Film without PbI_2_ for High Performance Solar Cell

**DOI:** 10.1038/srep35994

**Published:** 2016-10-27

**Authors:** Uisik Kwon, Md Mehedi Hasan, Wenping Yin, Dasom Kim, Na Young Ha, Soonil Lee, Tae Kyu Ahn, Hui Joon Park

**Affiliations:** 1 Division of Energy Systems Research, Ajou University, Suwon 16499, Korea; 2 Department of Energy Science, Sungkyunkwan University, Suwon 16419, Korea; 3 Department Electrical and Computer Engineering, Ajou University, Suwon 16499, Korea

## Abstract

In CH_3_NH_3_PbI_3_-based high efficiency perovskite solar cells (PSCs), tiny amount of PbI_2_ impurity was often found with the perovskite crystal. However, for two-step solution process-based perovskite films, most of findings have been based on the films having different morphologies between with and without PbI_2_. This was mainly due to the inferior morphology of pure perovskite film without PbI_2_, inevitably produced when the remaining PbI_2_ forced to be converted to perovskite, so advantages of pure perovskite photoactive layer without PbI_2_ impurity have been overlooked. In this work, we designed a printing-based two-step process, which could not only generate pure perovskite crystal without PbI_2_, but also provide uniform and full surface coverage perovskite film, of which nanoscale morphology was comparable to that prepared by conventional two-step solution process having residual PbI_2_. Our results showed that, in two-step solution process-based PSC, pure perovskite had better photon absorption and longer carrier lifetime, leading to superior photocurrent generation with higher power conversion efficiency. Furthermore, this process was further applicable to prepare mixed phase pure perovskite crystal without PbI_2_ impurity, and we showed that the additional merits such as extended absorption to longer wavelength, increased carrier lifetime and reduced carrier recombination could be secured.

Recently, organometal trihalide perovskite materials having composition ABX_3_ (*e.g.* A = Cs^+^, CH_3_NH_3_^+^ (methylammonium, MA), or HC(NH_2_)_2_^+^ (formamidinium, FA); B = Pb or Sn; X = I, Br or Cl) have been investigated extensively for use as light-absorbing material in solar cells because of their unique properties such as direct optical bandgap, broadband light absorption, bipolar transport, and long carrier diffusion length. Since the first report about perovskite solar cells (PSC) having 3.81% power conversion efficiency (PCE) by Kojima *et al*. in ref. 1, which triggered intensive research in the development of PSC, remarkable enhancement in power conversion efficiency (PCE) reaching 20% has been achieved during past several years^2^,^3^,^4^.

In conventional silicon-based p-n junction photovoltaic (PV) devices, the pure crystal structure in photoactive layer has been known to be advantageous to efficient charge transport and reduced exciton quenching for high efficiency solar cell. However, in MAPbI_3_-based PSC showing high efficiency, tiny amount of residual PbI_2_ impurity was often found with the perovskite crystal phase, even though the equimolar composition of organic (MAI) and inorganic (PbI_2_) components was utilized to fully convert them to perovskite crystal^3^,^5^,^6^,^7^,^8^,^9^,^10^,^11^,^12^,^13^,^14^. Therefore, various approaches have been reported to find out if perovskite crystal with PbI_2_ impurity would be advantageous to the performance of PSC or not. However, in general, the crystalline structure and nanoscale morphology of perovskite photoactive layers are significantly influenced by their deposition methodology^15^,^16^,^17^,^18^,^19^,^20^,^21^,^22^, and therefore those reports should be individually interpreted depending on their growth mechanism.

Chen *el al.* reported an approach to produce pure MAPbI_3_ film by treating as-deposited PbI_2_ film with MAI vapor for several hours, from which PbI_2_ component could be reversibly regenerated when annealed at 150 °C^5^,^6^. They showed that the regenerated PbI_2_ from the pure MAPbI_3_ crystal structure by annealing was helpful to passivate grain boundary (GB) between crystal domains, consequently improving their device performances due to the reduced recombination^6^. Similarly, Zhang *et al*. investigated the role of PbI_2_ in their perovskite film, grown by spin-casting hydrohalide deficient PbI_2_·xHI (x = 0.9~1) precursor under MA vapor atmosphere. Using their process, the proper amount of PbI_2_ nanoplates could be located at the GB, which could consequently provide a longer photoluminescence (PL) life time and enhanced open-circuit voltage (*V*_oc_) to their PSC^7^.

As for the one-step solution casting-based perovskite films, Wang *et al*. studied their perovskite films using the time-resolved femtosecond transient absorption (fs-TA) spectroscopy and the slower relaxation rates were obtained from the perovskite film with PbI_2_, which proved the passivation effect of PbI_2_ on the perovskite GB^8^. Similarly, the positive effects of excess PbI_2_, added to intentionally exceed the equimolar composition of the components, were also shown, which were the reduction of ionic defect migrations^9^ and trap density close to the perovskite/TiO_2_ interface^3^, consequently improving their device performances. In contrast, there was a report, in which pure MAPbI_3_ crystal structure without PbI_2_, prepared by an one-step spin-coating of the solution of MAI and PbI_2_ mixture in *N*,*N*-dimethylformamide (DMF) with hydriiodic acid (HI) additive, was advantageous to decrease hysteresis as well as to improve the device performances^10^. These conflicting results may be because the amount, form, morphology or location of the residual PbI_2_ could vary in perovskite films, prepared by different processing conditions during the one-step solution casting.

The growth mechanism of the perovskite crystal from the two-step sequential solution processes (*e.g.* dip-coating or spin-casting MAI on the spin-casted PbI_2_), known to follow dissolution and recrystallization procedures at the interface between liquid and solid generally around the PbI_2_ surface, is different to that from the one-step mixed solution-based processes, known to follow interlayer diffusion^11^. Therefore, we can speculate that the effect of residual PbI_2_ in photoactive layers during the device operation would be also different depending on their fabrication methods. Cao *et al*.^12^ and Lee *et al*.^11^ individually showed that PSCs having remnant PbI_2_ in two-step solution-based photoactive layers produced higher PCE, but the inferior performances, obtained from the pure perovskite phase-based devices, mainly originated in the non-uniformly grown perovskite films having over-grown needle-like structures due to longer dipping time to force the remaining PbI_2_ converted^12^ or coarse nanomorphology having a lot of pinholes due to the recrystallization during their film formation^11^. Similarly, in the work by Wang *et al*., the perovskite film without PbI_2_, which provided inferior solar cell performances, was prepared by casting MAI on the coarse PbI_2_ layer, consequently inducing the low surface coverage perovskite film^13^. Therefore, to clearly verify the effect of PbI_2_ in the two-step process-based PSC, the morphological discrepancy between perovskites with and without PbI_2_ should be eliminated, first. Furthermore, Lee *et al*. showed the possibility that the higher conduction band of PbI_2_ than that of perovskite could block the electron injection from perovskite to TiO_2_^11^, and Liu *et al*. proved that the residual PbI_2_ induced an intrinsic instability to the film under illumination condition, consequently degrading the film^14^. Therefore, the merits of PbI_2_ in two-step solution process-based perovskite, are still ambiguous.

In this work, we designed a printing-based two-step solution process, which could not only generate pure perovskite crystal without residual PbI_2_, but also provide uniform and full surface coverage perovskite film, of which the nanoscale morphology was comparable to that prepared by conventional two-step solution processes (such as spin-casting and dip-coating) generally containing a certain amount of remnant PbI_2_, to find out the advantages of pure perovskite on the performance of two-step sequential process-based PSC. Therefore, it is proper to have in-depth understanding of the advantages of pure perovskite crystal in two-step solution process-based PSC after excluding the morphology-driven effects. The information about the crystallinity and the morphological characteristics of perovskite films was investigated by X-ray diffraction (XRD) patterns and scanning electron microscopy (SEM), respectively. Our results showed that, in two-step solution process-based PSC, pure perovskite photoactive layer had better photon absorption and longer carrier life time, revealed by UV-Vis spectroscopy and time-resolved photoluminescence (TRPL), respectively, leading to superior photocurrent generation with higher PCE. Furthermore, this process was further applicable to prepare mixed phase pure perovskite crystal without PbI_2_ impurity, and we showed that partial replacement of MA cation of pure MAPbI_3_ with FA cation using the printing process, not degrading the high crystallinity without PbI_2_ impurity and forming α-phase (MA)_x_(FA)_1−x_PbI_3_, was beneficial to broadening the absorption wavelength range with the additional increase of the carrier life time and the reduction of charge recombination, proved by UV-Vis, TRPL, and transient photovoltage (TPV), respectively.

## Results and Discussion

**Figure 1:**
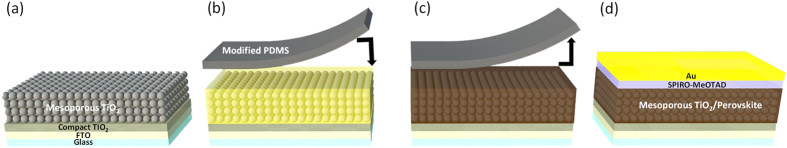
Schematic of the solar cell fabrication having pure perovskite crystal without residual PbI_2_: (**a**) electron transport layer (blocking and mesoporous TiO_2_) formation (FTO refers to fluorine-doped tin oxide); (**b**) applying MAI solution on PbI_2_-casted TiO_2_ layer (PDMS refers to polydimethylsiloxane); (**c**) perovskite crystal photoactive layer formation during the solvent evaporation under pressure; and (**d**) Au electrode deposition on top of the hole transport layer (Spiro-MeOTAD), casted on perovskite layer, after removing the gas-permeable stamp.

To prepare two-step process-based pure perovskite film having uniform nanomorphology, soft printing-based process was designed as shown in Fig. 1. On a FTO-coated glass, compact TiO_2_ and mesoporous (mp) TiO_2_ layers were sequentially added, and PbI_2_ dissolved in DMF was deposited over the mp TiO_2_ layer. On top of the dried PbI_2_ layer, MAI solution in 2-propanol was dispensed and capped with a gas-permeable silicone film enabling solvent evaporation, such as polydimethylsiloxane (PDMS), to which a slight pressure was applied. The solidified perovskite layer remained on the TiO_2_ substrate after removal of the silicone film. Because a shear stress applied to the solution across the whole depth between substrate and silicone film is much more effective than that between a substrate and an air surface (*e.g.* spin-casting), which decreases from the substrate to be zero at the air interface^23^,^24^,^25^, it is expected that MAI during this process can be more efficiently diffused to PbI_2_ in mp TiO_2_ template than that during the conventional spin-casting process. Moreover, the solvent dwelling time can be adjusted by controlling the applied pressure or utilizing silicone films having different gas-permeability^23^,^24^, thus this approach is advantageous to optimize the conversion of the components (MAI and PbI_2_) to perovskite crystal, different to the conventional spin-casting. Even though dipping process can be utilized to freely control the solvent dwelling time in two-step solution process, it does not have any extendibility to continuous process such as roll-to-roll. In our former work, we have shown that this type of printing-based casting process could be extended to the continuous roll-to-roll process, by which a three-inch-wide uniform large area polymer bulk-heterojunction photoactive layer for organic solar cell was demonstrated^23^,^24^. The extendibility of this printing-based process to the large area continuous fabrication is highly promising to the future high efficiency PSC technology. Detailed procedure of solar cell fabrication is described in Experimental Section.

**Figure 2:**
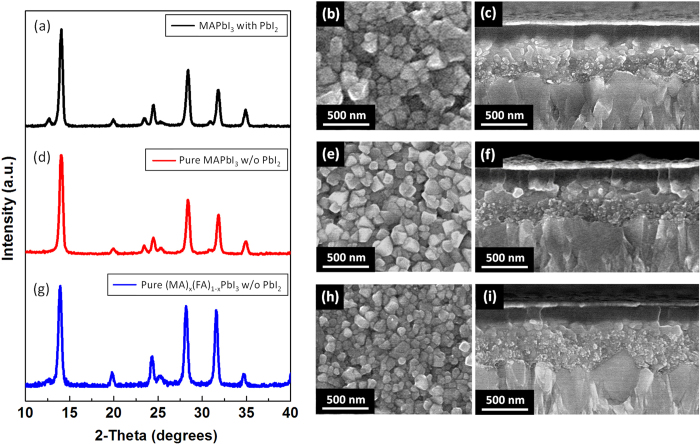
Perovskite, MAPbI_3_, with PbI_2_: (**a**) XRD patterns, (**b**) top-view SEM image of perovskite film, and (**c**) cross-section SEM image of complete device structure. Pure perovskite, MAPbI_3_, without PbI_2_: (**d**) XRD patterns, (**e**) top-view SEM image of perovskite film, and (**f**) cross-section SEM image of complete device structure. Pure perovskite, (MA)_x_(FA)_1−x_PbI_3,_ without PbI_2_: (**g**) XRD patterns, (**h**) top-view SEM image of perovskite film, and (**i**) cross-section SEM image of complete device structure. As for pure MAPbI_3_ and (MA)_x_(FA)_1−x_PbI_3_ without PbI_2_, 10 mg·ml^−1^ of MAI solution and 8 + 2 mg·ml^−1^ of MAI + FAI mixed solution were utilized for printing process, respectively.

The formation of pure perovskite crystal and its morphology were investigated by XRD patterns and SEM images. Figure 2d confirmed that pure perovskite crystal phase without PbI_2_ was successfully prepared by printing-based process using 10 mg·ml^−1^ MAI solution, of which the existence could be identified by the peak at 2*θ* = 12.6°, attributed to the (001) lattice planes of hexagonal (2H polytype) PbI_2_^5^,^6^,^7^,^8^,^9^,^10^,^11^,^12^,^13^,^14^. In contrast, the small trace of PbI_2_ peak was still detected from the XRD patterns of the two-step spin-casting-based perovskite film (Fig. 2a), similar to the literatures^11^,^12^,^13^,^14^. SEM images in Fig. 2e,f showed that compact and fully covered nanocube-shaped perovskite morphology could be generated from the printing-based process, which was comparable to the morphology, prepared by the two-step spin-casting-based process (Fig. 2b,c). Therefore, our approach is proper to study the effect of pure crystallinity in the two-step process-based perovskite photoactive layer on the performances of the PSCs by excluding the structural discrepancy effect, which has induced the performance variation between the perovskite films with and without PbI_2_ in the former reports^11^,^12^,^13^,^14^. Meanwhile, at the other MAI concentrations for the printing process, lower or higher than 10 mg·ml^−1^, neither uniform nanoscale morphology nor pure perovskite crystal were found (Figure S1). Thus, the perovskite films from those MAI concentrations for the printing process were not suitable for our study to investigate the effect of pure perovskite crystal on the performances of PSCs. The effect of MAI concentration on the crystallinity and morphology of the printed perovskite films will be discussed in detail later with the performances of PSCs. As for the spin-casting-based perovskite, the film prepared using 10 mg·ml^−1^ concentration of MAI, which showed the best performances (data not shown), was selected as a comparison to the printed pure perovskite film. In the spin-casted perovskite films, the residual PbI_2_ was always detected regardless of MAI concentration as reported in the literatures^11^,^12^,^13^,^14^.

**Figure 3:**
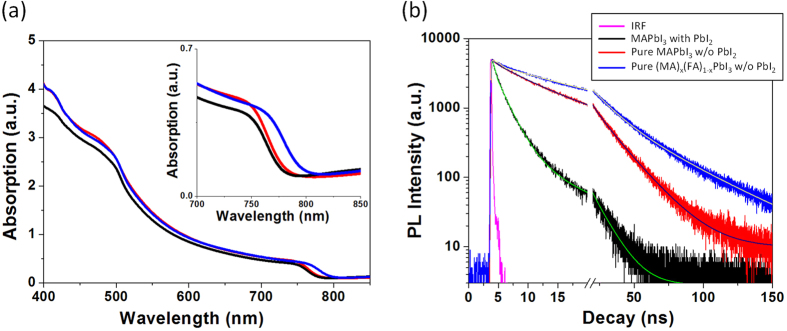
(**a**) UV-Vis absorbance and (**b**) Time-resolved photoluminescence (TRPL) spectra of perovskite layers on quartz, taken at peak emission wavelength (770 nm), excited at 670 nm (1 MHz) from perovskite side. Black, red and blue colors represent perovskite, MAPbI_3_, with PbI_2_, pure perovskite, MAPbI_3_, without PbI_2_ and pure perovskite, (MA)_x_(FA)_1−x_PbI_3,_ without PbI_2_, respectively. As for pure MAPbI_3_ and (MA)_x_(FA)_1−x_PbI_3_ without PbI_2_, 10 mg·ml^−1^ of MAI solution and 8 + 2 mg·ml^−1^ of MAI + FAI mixed solution were utilized for printing process, respectively.

The pure perovskite film showed the enhanced photon absorption, compared to the film with residual PbI_2_. As shown in UV-Visible spectra (Fig. 3a), the perovskite film with pure perovskite crystal had obviously higher absorption than the film having residual PbI_2_ impurity over the whole wavelength range in the spectrum. Because the thicknesses of both perovskite films were almost identical (Fig. 2c,f), the enhanced absorption was due to the increased crystallinity. In addition, the improved crystallinity of perovskite film without residual PbI_2_ provided the longer carrier life time as analyzed by the TRPL decay of Quartz/perovskite sample. The decay curves of each sample were convoluted using bi-exponential functions as plotted in Fig. 3b. According to bi-exponential fitting, the perovskite with the residual PbI_2_ showed a medium component of 2.12 ns and a long component of 11.32 ns (Table S1), which should originate in the bimolecular recombination in perovskite active layer and free carrier recombination in the radiative channel, respectively^6^,^26^. This result also showed good consistency with the documented literatures^27^,^28^. In contrast, the pure perovskite film without remnant PbI_2_ showed medium component of 6.24 ns and long component of 18.56 ns, representing that pure perovskite film had longer carrier life time with low trap density due to a better quality of perovskite crystal, advantageous to superior charge generation by reducing the recombination within the photoactive layer.

**Figure 4:**
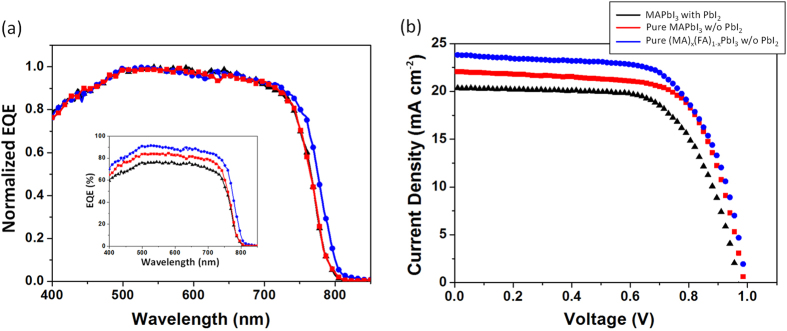
Device performances depending on the condition of perovskite photoactive layer. Color of line and symbol are as in Fig. 3. (**a**) External quantum efficiency (EQE). (**b**) *J*-*V* plots (All data were measured at AM 1.5 G with an intensity of 100 mW·cm^−2^.). Perovskite solar cell characteristics are summarized as follows: perovskite, MAPbI_3_, with PbI_2_ [*J*_sc_ = 20.4 mA·cm^−2^, *V*_oc_ = 0.97 V, *FF* = 0.66, PCE = 13.0%]; pure perovskite, MAPbI_3_, without PbI_2_ [*J*_sc_ = 22.0 mA·cm^−2^, *V*_oc_ = 0.99 V, *FF* = 0.69, PCE = 15.0%]; pure perovskite, (MA)_x_(FA)_1−x_PbI_3_, without PbI_2_, [*J*_sc_ = 23.9 mA·cm^−2^, *V*_oc_ = 0.99 V, *FF* = 0.66, PCE = 15.6%]. As for pure MAPbI_3_ and (MA)_x_(FA)_1−x_PbI_3_ without PbI_2_, 10 mg·ml^−1^ of MAI solution and 8 + 2 mg·ml^−1^ of MAI + FAI mixed solution were utilized for printing process, respectively.

We fabricated solar cells using the perovskite photoactive layers with and without PbI_2_. On top of the photoactive layer, prepared by two-step-based processes on mp-TiO_2_, 2,2′,7,7′-Tetrakis-(N,N-di-4-methoxyphenylamino)-9,9′-spirobifluorene (Spiro-MeOTAD) was casted as a hole transport layer (HTL) using chlorobenzene, an orthogonal solvent to the perovskite layer. Finally, the thermal deposition of Au completed the device fabrication. The short-circuit current density (*J*_sc_), *V*_oc_, fill factor (*FF*), and PCE of the devices under AM 1.5G simulated sunlight (at 100 mW·cm^−2^ intensity) are summarized in Fig. 4. These parameters are average values of the *J*-*V* characteristics obtained by scanning those in forward and reverse directions at a 0.05 V·s^−1^ scan rate. The detailed solar cell data about hysteresis with both forward and reverse scans are represented in Figure S2. As shown in Fig. 4, the solar cells fabricated by the pure perovskite (PCE = 15.0%) outperformed those by the perovskite with residual PbI_2_ (PCE = 13.0%), and the improved PCE was mainly from *J*_sc_ (from 20.4 to 22.0 mA·cm^−2^) and *FF* (from 0.66 to 0.69), believed to originate in the increased light absorption and carrier life time of the pure perovskite crystal. Meanwhile, as mentioned earlier, the morphologies of the printed perovskite films, prepared by the MAI concentrations, lower or higher than 10 mg·ml^−1^, were not uniform and the residual PbI_2_ also remained (Figure S1). At 5 mg·ml^−1^, lower than the optimum concentration (10 mg·ml^−1^), the size of nanocube were found to be much larger and disconnected each other, causing non-uniform and discrete perovskite film that resulted in the less surface coverage and capping of underlying TiO_2_ film (Figure S1b), and a strong crystalline PbI_2_ peak was observed at 12.6° (Figure S1a), suggesting that this concentration of MAI was not sufficient for the complete conversion of PbI_2_ into perovskite. When a HTL is deposited over this discrete perovskite film, direct contact between HTL and electron transporting layer (ETL) is more likely to form the shunt pathways, resulting in the degraded performance of device. Because of uncompleted conversion of PbI_2_ into perovskite and the shunt pathway created by the direct contact between ETL and HTL, the performance of the cell fabricated using 5 mg·ml^−1^ of MAI was found to be extremely poor. *V*_oc_, *J*_*sc*_ and *FF* of such devices were found to be 0.73 V, 6.9 mA·cm^−2^ and 0.39 respectively, which resulted in the PCE to be 2.0% (average values of forward and reverse scan). Increasing the MAI concentration can improve the interconnectivity of the perovskite nanocubes while decreasing their sizes (Figure S1b,e,h,k). At the MAI concentration of 10 mg·ml^−1^, the uniform nanomorphology with the full conversion of PbI_2_ to the pure perovskite was obtained as explained, however, over that concentration (*e.g.* 15 mg·ml^−1^ and 20 mg·ml^−1^ of MAI), the size of perovskite nanocube further decreased (Figure S1h,k) and small PbI_2_ peak was shown again (Figure S1g,j), which was likely due to the decomposition of MAPbI_3_^29^. Especially, at the highest concentration of MAI (20 mg·ml^−1^), a peak around 2*θ* = 11.5°, related to the low-dimensional perovskite structures (LDPs) such as 0D (quantum dot), 1D (chain) and 2D (sheet) structures, known to be found in MAI-rich film^30^,^31^, was generated, and the performances eventually decreased to 6.8% PCE (*V*_oc_ = 0.89 V, *J*_*sc*_ = 11.4 mA·cm^−2^ and *FF* = 0.66; average values of forward and reverse scan). The performance variation of the printed PSCs according to the MAI concentration is summarized in Figure S3.

It has been reported that FAPbI_3_ having formamidinium cation (HC(NH_2_)_2_^+^, FA, ionic radius: 1.9–2.2 Å), of which the size is slightly larger than that of methylammonium cation (CH_3_NH_3_^+^, MA, ionic radius: 1.8 Å), has a smaller bandgap (~1.48 eV) than MAPbI_3_ due to the lowered symmetry, providing the increased absorption edge of photon reaching to 840 nm^32^,^33^,^34^. However, black perovskite-type polymorph (α-phase) FAPbI_3_, which has this advantageous panchromatic property, is stable at the high temperature over 160 °C and it is easily turned into yellow non-perovskite-type δ-phase FAPbI_3_, which has wide bandgap and inferior charge-transport property due to the linear chain-like [PbI_6_] octahedron structure with edge sharing, at the ambient condition^33^,^34^. Jeon *et al*. demonstrated that α-phase FAPbI_3_ perovskite could be stabilized by adding both MA^+^ cations and Br^−^ anions to FAPbI_3_, which formed (FAPbI_3_)_0.85_(MAPbBr_3_)_0.15_, leading to 17.3% PCE^2^, however, their approach was not only based on the mixed halide system, composed of I and Br, but also they utilized the one-step solution casting process. As for the two-step solution-based process, Pellet *et al*. successfully represented that α-phase FAPbI_3_ perovskite could be stabilized by gradually replacing MA with FA cation to form (MA)_x_(FA)_1−x_PbI_3_ (x = 0–1), of which two cations were inserted in the same lattice frame^35^. They prepared their FAPbI_3_-containing mixed phase photoactive layer by dipping the spin-casted PbI_2_ into the MAI and FAI mixture solution. Even though they showed the improved performances from 12.0% to 13.4% PCE after introducing FA cation, the residual PbI_2_ was still detected in their photoactive layer, which meant that pure perovskite crystal was not formed. In contrast, in our mixed phase photoactive layer, prepared by printing MAI and FAI mixture solution (8 + 2 mg in 1 ml 2-propanol) on the PbI_2_ layer, not only α-phase (MA)_x_(FA)_1−x_PbI_3_ was confirmed but also no trace of PbI_2_ was found from the XRD patterns (Fig. 2g), which meant pure perovskite crystal without residual PbI_2_ impurity was obtained. The pure perovskite crystal structure without PbI_2_ impurity was preserved to the film, prepared by 7 + 3 mg·ml^−1^ concentration of MAI + FAI solution (Figure S4). We also observed the decrease of diffraction angles about α-phase perovskite structure with the use of MAI and FAI mixture from 14.02, 19.94, 24.26, 28.36 and 31.84° (10 mg·ml^−1^ MAI case, Fig. 2d), to 13.88, 19.78, 24.26, 28.12 and 31.44° (8 + 2 mg·ml^−1^ of MAI + FAI case, Fig. 2g), respectively, which meant the expansion of the crystal lattice due to the insertion of larger size FA cation. Figure S5 shows that the diffraction angle gradually decreases with the increase of FAI content, and this indicates the formation of (MA)_x_(FA)_1−x_PbI_3_ mixed phase having two cations in the same lattice^35^. The XRD patterns of all (MA)_x_(FA)_1−x_PbI_3_ samples are shown in Figure S4.

**Figure 5:**
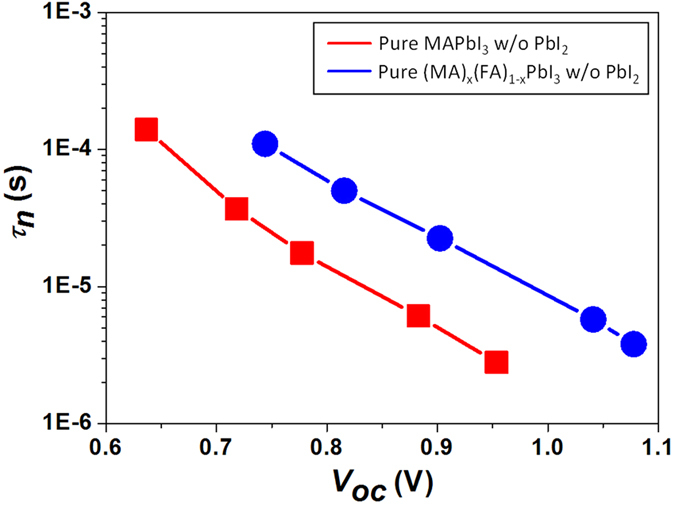
Recombination life times (*τ*_*n*_) measured by transient photovoltage (TPV) measurement of complete cells having pure perovskite layers without PbI_2_: Red and blue colors represent MAPbI_3_, prepared by printing 10 mg·ml^−1^ of MAI solution, and (MA)_x_(FA)_1−x_PbI_3_, prepared by printing 8 + 2 mg·ml^−1^ of MAI + FAI mixed solution, respectively.

Furthermore, uniform nanomorphologies with full surface coverage were confirmed from our (MA)_x_(FA)_1−x_PbI_3_ mixed perovskite films as shown in SEM images (Figs 2h,i and S4), and the prepared photoactive layer (8 + 2 mg·ml^−1^ of MAI + FAI) obviously showed the increased band edge as represented in absorption spectra (Fig. 3a), which contributed to the additional photocurrent generation at the longer wavelength range as shown in external quantum efficiency (EQE) (Fig. 4a). Moreover, the average carrier life time in the photoactive layer (8 + 2 mg·ml^−1^ of MAI + FAI), calculated from TRPL data, was further increased to the maximum values, 14.75 ns and 42.22 ns for the medium and long components of bi-exponential function of TRPL spectra, respectively. They were 2.12 ns and 11.32 ns in the MAPbI_3_ perovskite film with PbI_2_ and 6.24 ns and 18.56 ns in the pure MAPbI_3_ perovskite film without PbI_2_ as mentioned earlier (Fig. 3b and Table S1). The recombination life time of charge carrier (*τ*_*n*_) in the PSC devices could be further evaluated by TPV technique and the PSC having the mixed phase pure perovskite crystal, composed of MA and FA mixed cations without PbI_2_, showed the longer recombination life time than that having the pure MAPbI_3_ crystal structure without PbI_2_ (Fig. 5). Consequently, the increased carrier life time and reduced recombination in pure (MA)_x_(FA)_1−x_PbI_3_ improved their overall EQE in whole absorption wavelength range, compare to pure MAPbI_3_ (inset of Fig. 4a), even with the similar amount of photon absorption (Fig. 3a), and their corresponding device performances (8 + 2 mg·ml^−1^ of MAI + FAI) were improved to give 15.6% PCE (*V*_oc_ = 0.99 V, *J*_*sc*_ = 23.9 mA·cm^−2^ and *FF* = 0.66; average values of forward and reverse scans).

## Conclusions

In two-step solution process-based MAPbI_3_ photoactive layer, we revealed that pure perovskite crystal structure without residual PbI_2_ was advantageous to increase the photon absorption and the carrier life time, consequently improving *J*_*sc*_ and *FF* of PSCs, if the uniform and dense perovskite nanomorphology could be preserved without residual PbI_2_. This study was possible by designing a printing-based process that could produce not only highly pure perovskite thin film without remnant PbI_2_ but also uniform nanomorphology with full surface coverage, by which we could exclude the morphological discrepancy between the perovskite films with and without PbI_2_ for the reasonable comparison. Moreover, this process was further applicable to prepare (MA)_x_(FA)_1−x_PbI_3_ pure perovskite crystal without PbI_2_ impurity, of which two MA and FA cations were inserted in the same lattice frame, and we confirmed that the additional merits such as the extended absorption range to longer wavelength, the increased carrier life time and the reduced charge carrier recombination could be secured.

## Methods

### Device fabrication

Devices were fabricated on fluorine-doped tin oxide(FTO)-coated glass. The region of FTO under the anode contact was etched using 35% HCL and zinc powder. Substrates were then cleaned subsequently in acetone, isopropanol, deionized water and oxygen plasma. About 50 nm of hole blocking layer of compact TiO_2_ was deposited by spin-casting twice a 0.15 M solution of titanium diisopropoxide bis(acetylacetonate) (75% in 2-propanol, Sigma-Aldrich) and annealed at 500 °C for 30 min. The substrates were treated with 0.04 M of TiCl_4_ aqueous solution. About 250 nm thick mesoporous TiO_2_ layer was then deposited over the hole blocking layer by spin-casting a solution of commercial TiO_2_ paste (Dyesol 30NRD) in anhydrous ethanol and annealed at 500 °C at 30 min. Then the substrates were subsequently cleaned with deionized water and ethanol and annealed at 500 °C for 30 min. 461 mg/ml PbI_2_ (99.9% Sigma-Aldirch) solution in DMF was stirred for 8 hours at 70 °C and then was spin-casted (4000 rpm for 40 seconds) over the substrate, followed by annealing at 70 °C for 5 min. In case of printing devices, MAI or mixture of MAI and FAI in 2-propanol having the required concentration was deposited by squeezing the solution between a gas-permeable silicone film^36^ and PbI_2_/mp-TiO_2_/bl-TiO_2_/FTO substrate. After printing, the devices were demolded and annealed at 110 °C for 10 min. In case of two-step deposition procedure, MAI in 2-propanol was spin-casted over dry PbI_2_ at speed of 2000 rpm for 30 seconds followed by annealing at 110 °C for 10 min. About 150 nm thick hole transporting layer was deposited by spin-casting the solution containing 35 mg Spiro-MeOTAD mixed with 1 ml of chlorobenzene and additives of 4.4 mg of lithium bis(trifluoromethanesulfonyl)-imid, 14 μl of 4-tert-butylpyridine and 17.5 μl of acetonitrile. Finally about 70 nm of gold was thermally evaporated to complete the devices.

### Device characterization

A solar simulator (PEC-L01, Peccell Technologies, Inc.) with an AM 1.5G filter was used to provide 100 mW·cm^−2^ of illumination on the PV cells, with the intensity calibrated using a Si photodiode. *J*–*V* characteristics were obtained using an Ivium technology Ivium compactstat by scanning the *J*-*V* curves at a 0.05 V·s^−1^ scan rate. The incident-photon-to-electron conversion efficiency (IPCE) was measured under short-circuit conditions using ABET Technology 10500 solar simulator as the light source and a SPECTRO Mmac-200 as the light solution. The differences between the short-circuit current densities obtained by *J*-*V* curves and those calculated by EQE signal are within 10% (Calculated *J*_sc_ values from EQE signals are 18.6, 20.3 and 22.5 mA·cm^−2^ for MAPbI_3_ with PbI_2_, pure MAPbI_3_ w/o PbI_2_, and pure (MA)_x_(FA)_1−x_PbI_3_ w/o PbI_2_, respectively). UV–visible absorption spectra were recorded with a Jasco V760 UV-Vis NIR spectrophotometer in the 400–850-nm wavelength range at room temperature. XRD patterns of the prepared films were obtained using a Rigaku Ultima III high-resolution X-ray diffractometer. SEM images were obtained by Hitachi S-4800. Time-resolved photoluminescence (TRPL) curves were recorded using a commercial Time-Correlated Single Photon Counting (TCSPC) system (FluoTim 200, PicoQuant)^37^. Samples were photoexcited by picosecond diode laser of 670 nm (LDH-P-C-670, PicoQuant) with a variable repetition rate (1 MHz). The emitted PL was spectrally dispersed with monochromater (ScienceTech 9030) for each PL signal and was collected by a fast photon multiplier tube (PMT) detector (PMA 182, PicoQuant GmbH) with a magic angle (54.7°) arrangement. The incident angle of excitation pulse was set to be about 30° with respect to the sample. The resulting instrumental response function was about 160 ps in full-width-half-maximum. And all of the signals were measured at the emission peak (770 ± 5 nm) for perovskite. In addition, a cut-off filter (FF01-692 nm, Semrock) was applied to block the remaining scattering. Transient photovoltage (TPV) decay measurement was performed using a nanosecond laser (pulsed 10 Hz, NT342A-10, EKSPLA) pumped OPO pulse of 550 nm and a Xe lamp (continuous wave, 150 W, Zolix) as attenuated perturbation light pulse and a bias light source, respectively. The sample devices were electrically connected to a digital oscilloscope (500 MHz, DSO-X 3054A, Agilent) with BNC cables, and the input impedance was set to be 1 MΩ (Keithley 2001) for an open circuit condition. The bias light intensity was controlled by neutral density filters (from 0.0 to 1.0 sun) for various open circuit voltages (*V*_oc_). All the perovskite films, utilized to measure absorption, TRPL, and TPV, were the same as those for solar cell devices.

## Additional Information

**How to cite this article**: Kwon, U. *et al*. Investigation into the Advantages of Pure Perovskite Film without PbI_2_ for High Performance Solar Cell. *Sci. Rep.* **6**, 35994; doi: 10.1038/srep35994 (2016).

**Publisher’s note:** Springer Nature remains neutral with regard to jurisdictional claims in published maps and institutional affiliations.

## Supplementary Material

Supplementary Information
