# Corrigendum: Investigation into the Advantages of Pure Perovskite Film without PbI_2_ for High Performance Solar Cell

**DOI:** 10.1038/srep43979

**Published:** 2017-03-16

**Authors:** Ujwal Thakur, Uisik Kwon, Md Mehedi Hasan, Wenping Yin, Dasom Kim, Na Young Ha, Soonil Lee, Tae Kyu Ahn, Hui Joon Park

Scientific Reports 6: Article number: 35994; 10.1038/srep35994 published online: 10 27 2016; updated: 03 16 2017.

Ujwal Thakur was omitted from the author list in the original version of this Article. This has been corrected in the PDF and HTML versions of the Article, as well as the Supplementary Information file.

The author contributions section now reads:

H.J.P conceived the project and planned the experiments. U.T., U.K., M.M.H, W.Y. and D.K. performed the experiments. N.Y.H., S.L. and T.K.A. analyzed data. H.J.P wrote the manuscript. All authors discussed the results and commented on the manuscript. U.T. and U.K. contributed equally to this work.

