# Supplementary material for: Investigation into the Advantages of Pure Perovskite Film without PbI2 for High Performance Solar Cell
Source: Sci Rep. 2016 Oct 27;6:35994. doi: 10.1038/srep35994 (PMC5081521; doi:10.1038/srep35994)
Supplement: Supplementary Information [file srep35994-s1.pdf]

Supplemetary Information

Investigation into the Advantages of Pure Perovskite Film without PbI_2 for High Performance Solar Cell

Uisik Kwon[1], Md Mehedi Hasan[1], Wenping Yin[2], Dasom Kim[2], Na Young Ha[1], Soonil Lee[1], Tae Kyu Ahn[2], and Hui Joon Park[1,3,*]

[1]Division of Energy Systems Research, Ajou University, Suwon 16499, Korea
[2]Department of Energy Science, Sungkyunkwan University, Suwon 16419, Korea
[3]Department Electrical and Computer Engineering, Ajou University, Suwon 16499, Korea

*Correspondence

Hui Joon Park, Division of Energy Systems Research, Department of Electrical and Computer Engineering, Ajou University, Worldcupro 206, Yeongtong-gu, Suwon, 16499, Korea
E-mail: huijoon@ajou.ac.kr / Tel: +82-31-219-2577 / Fax: +82-31-219-2208

KEYWORDS: perovskite solar cell, pure perovskite crystal, lead iodide, methylammonium lead iodide, formamidinium lead iodide

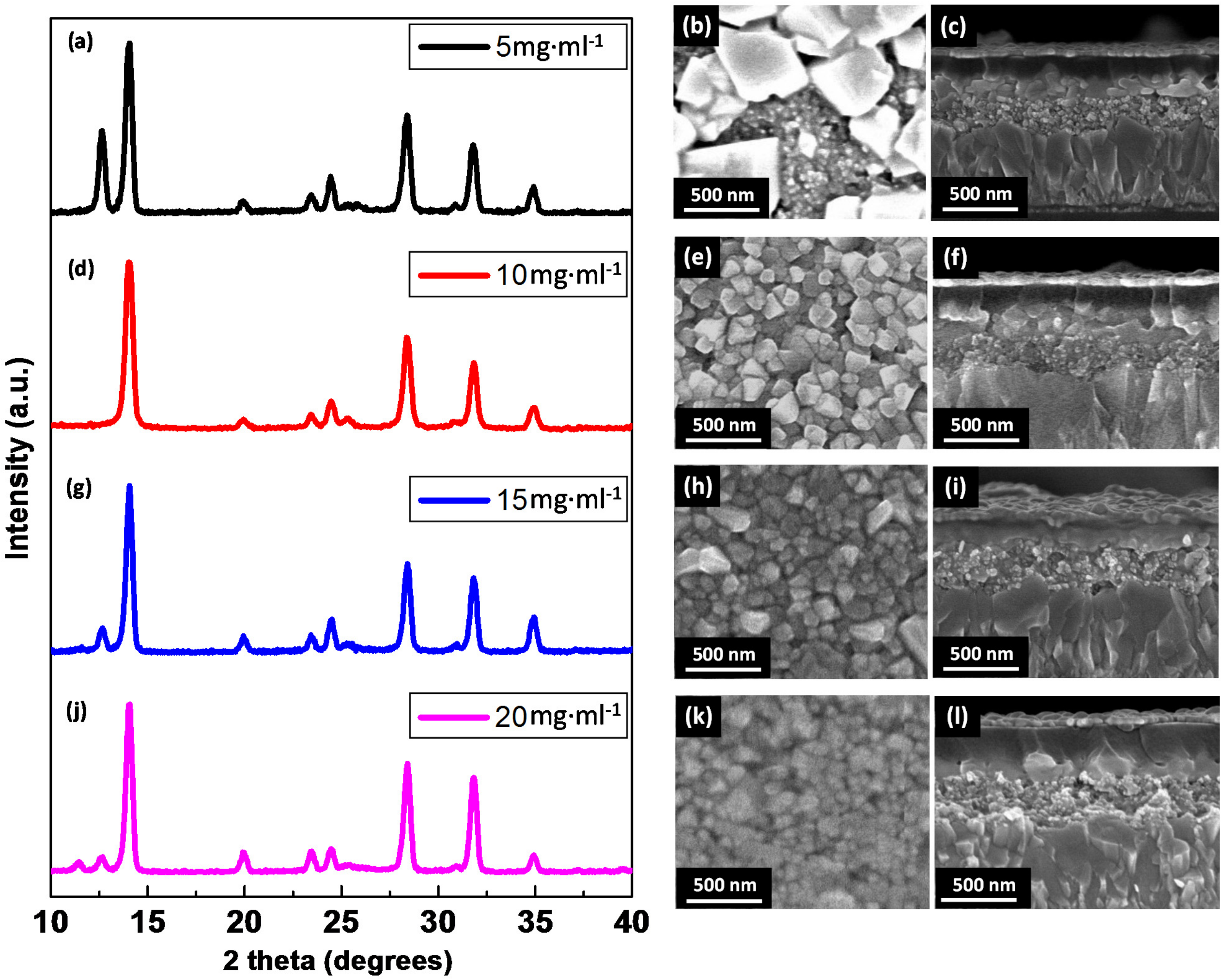

Figure S1. XRD patterns (a, d, g, j) and SEM top view images (b, e, h, k) of perovskite films, and SEM cross-section view images of complete devices (c, f, i, l), prepared by printing process using different MAI concentrations: (a-c) 5 mg·ml^{-1} MAI solution; (d-f) 10 mg·ml^{-1} MAI solution; (g-i) 15 mg·ml^{-1} MAI solution; (j-l) 20 mg·ml^{-1} MAI solution.

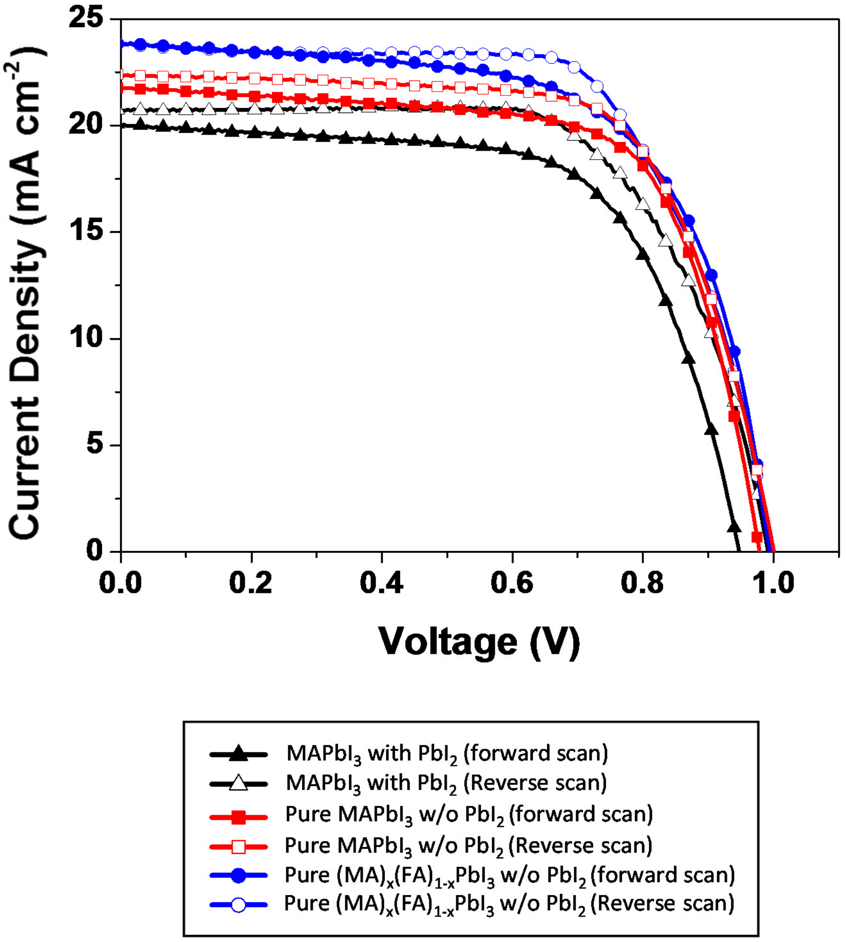

Figure S2. *J*-*V* plots (All data were measured at AM 1.5 G with an intensity of 100 mW·cm^{-2}.). Perovskite solar cell characteristics are summarized as follows: perovskite, $MAPbI_3$, with PbI_2 [Forward scan: J_{sc} = 20.0 mA·cm^{-2}, V_{oc} = 0.95 V, *FF* = 0.65, PCE = 12.4 %, Reverse scan: J_{sc} = 20.7 mA·cm^{-2}, V_{oc} = 0.99 V, *FF* = 0.67, PCE = 13.7 %]; pure perovskite, $MAPbI_3$, without PbI_2 [Forward scan: J_{sc} = 21.8 mA·cm^{-2}, V_{oc} = 0.98 V, *FF* = 0.69, PCE = 14.7 %, Reverse scan: J_{sc} = 22.3 mA·cm^{-2}, V_{oc} = 1.00 V, *FF* = 0.69, PCE = 15.3 %]; pure perovskite, $(MA)_x(FA)_{1-x}PbI_3$, without PbI_2, [Forward scan: J_{sc} = 23.9 mA·cm^{-2}, V_{oc} = 0.99 V, *FF* = 0.64, PCE = 15.2 %, Reverse scan: J_{sc} = 23.9 mA·cm^{-2}, V_{oc} = 0.99 V, *FF* = 0.68, PCE = 16.0 %]. As for pure $MAPbI_3$ and $(MA)_x(FA)_{1-x}PbI_3$ without PbI_2, 10 mg·ml^{-1} of MAI solution and 8+2 mg·ml^{-1} of MAI+FAI mixed solution were utilized for printing process, respectively.

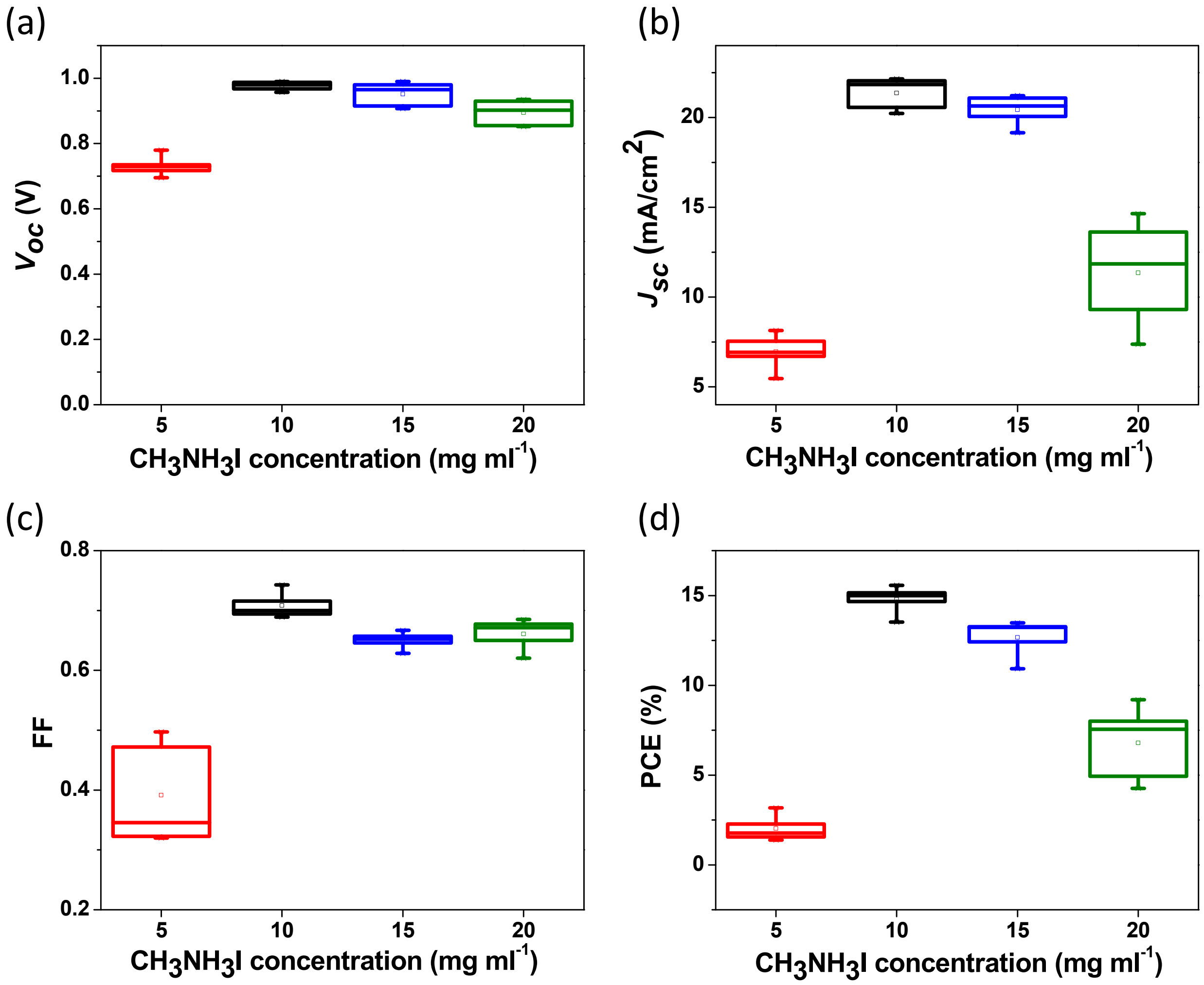

Figure S3. Summary of the performances of the printed perovskite solar cells ($MAPbI_3$) depending on the MAI solution concentration: (a) V_{oc}, (b) J_{sc}, (c) *FF* and (d) PCE. All the performance data were measured at AM 1.5 G with an intensity of 100 $mW \cdot cm^{-2}$ and average values of forward and reverse scan data.

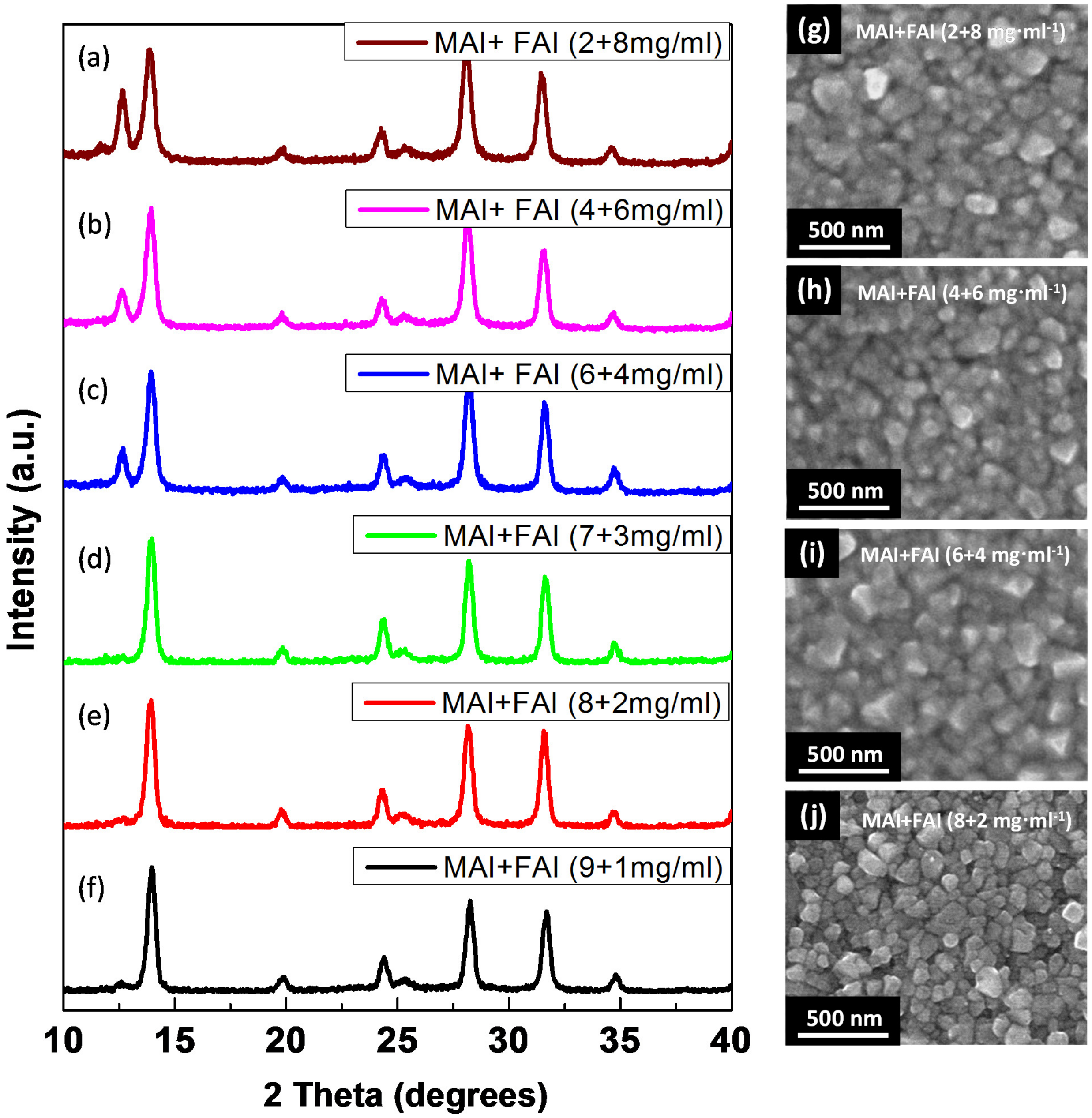

Figure S4. (a-f) XRD patterns and (g-j) SEM top view images of perovskite films, prepared by printing process using different MAI+FAI mixed solution concentration: (a,g) 2+8 mg·ml^{-1}; (b,h) 4+6 mg·ml^{-1}; (c,i) 6+4 mg·ml^{-1}; (d) 7+3 mg·ml^{-1}; (e,j) 8+2 mg·ml^{-1} ; (f) 9+1 mg·ml^{-1}.

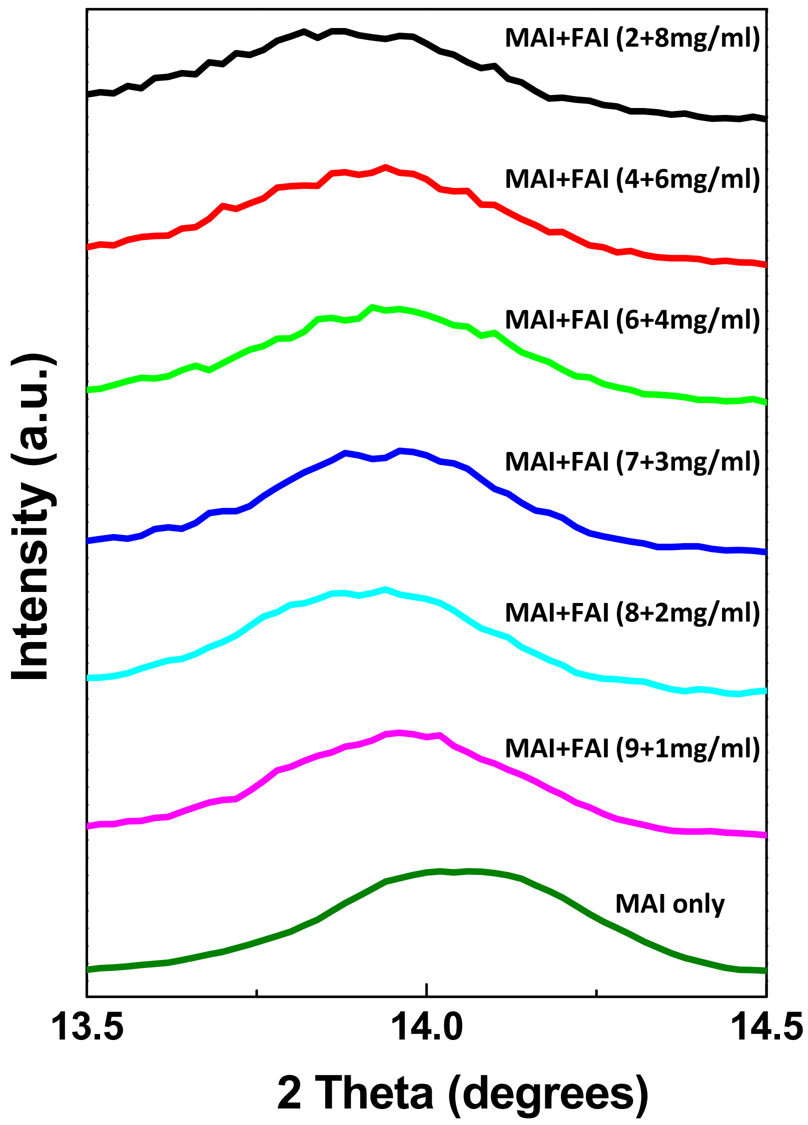

Figure S5. XRD patterns of perovskite films, prepared by printing process using different MAI+FAI mixed solution concentration. Magnified view of the region 13.5 – 14.5°. The peak at 14.02° (MAI only) shifts to lower reflection angles with FAI intercalation.

Table S1. Fitted parameters of TRPL (the values of the goodness-of-fit parameter (r^2) are all close to 1.0)

	A_1	τ_1(ns)	A_2	τ_2(ns)	τ_{avg}(ns)
$MAPbI_3$ with PbI_2	94.67 %	2.12	5.33 %	11.32	2.61
Pure $MAPbI_3$ w/o PbI_2	54.25 %	6.24	45.75 %	18.56	11.88
Pure $(MA)_x(FA)_{1-x}PbI_3$ w/o PbI_2	73.67 %	14.75	26.33 %	42.22	21.98

Where, $I(t) = A_1\exp(-t/\tau_1) + A_2\exp(-t/\tau_2)$
